# Supplementary material for: Causal Relationship between Aging and Anorexia Nervosa: A White-Matter-Microstructure-Mediated Mendelian Randomization Analysis
Source: Biomedicines. 2024 Aug 16;12(8):1874. doi: 10.3390/biomedicines12081874 (PMC11351342; doi:10.3390/biomedicines12081874)
Supplement: Supplementary file 1 [file biomedicines-12-01874-s001.zip › Supplementary Figure/Figure S8/Leaveoneout plot of MR analysis for TL to ubm-b-1998.pdf]

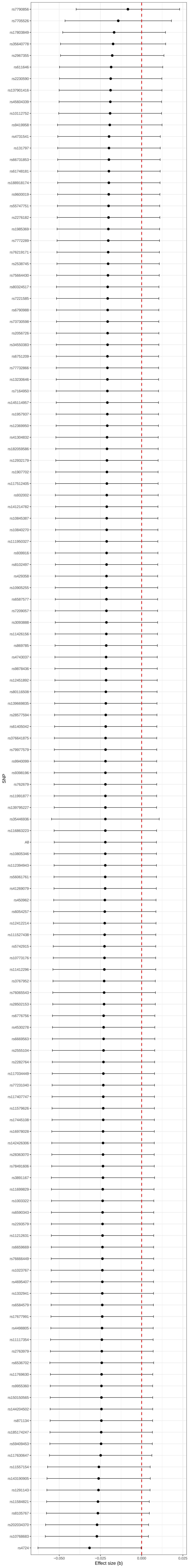

SNP
rs7790856
rs7705526
rs17803849
rs35640778
rs2967355
rs611646
rs2230590
rs137901416
rs45604339
rs10112752
rs9419958
rs4731541
rs131797
rs66731853
rs61748181
rs188918174
rs9600019
rs55747751
rs2276182
rs1985369
rs7772289
rs76219171
rs2538745
rs75664430
rs80324517
rs7221585
rs6790988
rs73730598
rs2056726
rs34550383
rs6751209
rs77732866
rs13230646
rs7164950
rs145114957
rs1957937
rs12369950
rs41304832
rs182059586
rs12932179
rs1907702
rs117512405
rs932002
rs141214782
rs10845387
rs10840270
rs111950327
rs939916
rs8102497
rs429358
rs10905255
rs6587577
rs7209057
rs3093888
rs11426156
rs869785
rs4743037
rs9878436
rs12451892
rs80116508
rs139669835
rs28577594
rs61405042
rs376641875
rs79977579
rs9940099
rs9398196
rs762679
rs11991877
rs139795227
rs35446936
rs116863223
All
rs10805346
rs112394943
rs56061761
rs41269079
rs450962
rs6054257
rs12412214
rs111527438
rs5742915
rs10773176
rs11412296
rs3767952
rs76065543
rs28502153
rs6776756
rs4530278
rs6669563
rs2555104
rs2282764
rs117034449
rs77231040
rs117407747
rs11579626
rs17445108
rs16978028
rs142426306
rs28363070
rs78491606
rs3891167
rs11699829
rs1003322
rs6590343
rs2293579
rs11212631
rs6659669
rs76666449
rs1023767
rs4695407
rs1332941
rs6584579
rs17677991
rs4498805
rs11117354
rs2763979
rs6536702
rs11769630
rs9955360
rs150150565
rs144204502
rs871134
rs185174247
rs59409453
rs117630647
rs11557154
rs143190905
rs1291143
rs11584821
rs8105767
rs202034370
rs10768683
rs4724
−0.050
−0.025
0.000
0.025
Effect size (b)
